# Inhibition of cell motility by troglitazone in human ovarian carcinoma cell line

**DOI:** 10.1186/1471-2407-7-216

**Published:** 2007-11-20

**Authors:** Yuh-Cheng Yang, Tsung-Chuan Ho, Show-Li Chen, Huei-Yi Lai, Ju-Yun Wu, Yeou-Ping Tsao

**Affiliations:** 1 Mackay Medicine, Nursing and Management College, Taipei, Taiwan; 2 Department of Medical Research, Mackay Memorial Hospital, Taipei, Taiwan; 3 School of Medicine, Taipei Medical University, Taipei, Taiwan; 4 Department of Microbiology, School of Medicine, National Taiwan University, Taipei, Taiwan; 5 Department of Microbiology and Immunology, The National Defense Medical Center, Taipei, Taiwan; 6 Department of Ophthalmology, Mackay Memorial Hospital, Taipei, Taiwan

## Abstract

**Background:**

Troglitazone (TGZ) is a potential anticancer agent. Little is known about the effect of this agent on cancer cell migration.

**Methods:**

Human ovarian carcinoma cell line, ES-2 cells were treated with various concentrations of TGZ. Cell migration was evaluated by wound-healing and Boyden chamber transwell experiments. PPARγ expression was blocked by PPARγ small interfering RNA. The effects of TGZ on phosphorylation of FAK, PTEN, Akt were assessed by immunoblotting using phospho-specific antibodies. The cellular distribution of paxillin, vinculin, stress fiber and PTEN was assessed by immunocytochemistry.

**Results:**

TGZ dose- and time-dependently impaired cell migration through a PPARγ independent manner. TGZ treatment impaired cell spreading, stress fiber formation, tyrosine phosphorylation of focal adhesion kinase (FAK), and focal adhesion assembly in cells grown on fibronectin substratum. TGZ also dose- and time-dependently suppressed FAK autophosphorylation and phosphorylation of the C-terminal of PTEN (a phosphatase). At concentration higher than 10 μM, TGZ caused accumulation of PTEN in plasma membrane, a sign of PTEN activation.

**Conclusion:**

These results indicate that TGZ can suppress cultured ES-2 cells migration. Our data suggest that the anti-migration potential of TGZ involves in regulations of FAK and PTEN activity.

## Background

Ovarian carcinoma is a leading cause of gynecologic cancer death [1]. The tumor spread into the peritoneal cavity is a hard-to-treat and frequent occurrence [2]. Morbidity and mortality rates due to disseminated ovarian carcinoma remain high [3,4]. Thus agents capable of preventing ovarian carcinoma metastasis may be of great therapeutic value.

Cancer metastasis involves cell proliferation, detachment of cells from extracellular matrix, invasion across basement membrane and vessel walls, and migration within extracellular matrix (ECM). Our current understanding of cell migration comes mainly from study of monolayer cultures of cancer cells. Cells attach to culture surface by forming focal adhesions (FAs) where ECM and integrins-associated membrane interact [5,6]. Cell migration involves assembly and disassembly of FAs and is stimulated extracellularly and initiated by intracellular signaling proteins located in FAs [5,6]. Focal adhesion kinase (FAK) is a non-receptor protein tyrosine kinase that is activated mainly in FAs and important in cell-ECM interactions that affect cell migration, proliferation, and survival [5-9]. Evidence indicates that overexpression of FAK is correlated with tumor progression and that FAK is significantly overexpressed in ovarian carcinoma [6,10,11]. Furthermore, immunohistochemical analysis of ovarian cancer samples reveals that enhanced FAK expression is correlated with ovarian carcinoma dissemination and poor prognosis [11].

The key event in FAK activation is autophosphorylation of Tyr397 [12]. It has been proposed that integrin clustering induced by cell spreading on matrix proteins promotes FAK autophosphorylation [9]. Autophosphorylation of FAK provides a binding site for Src family kinases that renders FAK phosphorylated at several other sites and leads to enhanced FAK activation [12,13]. Evidence indicates that FAK autophosphorylation promotes focal-complex assembly, and that many signaling and structural proteins such as Src kinases, vinculin, paxillin, and F-actin are recruited by the focal complex [5]. On the other hand, activated FAK can serve as an efficient scaffold protein for delivery of crucial molecules (such as calpain-2) to focal-adhesion sites that cause disassembly of FAs [5,14]. Therefore, FAK is a key molecule for controlling cell migration owing to its involvement in the regulation of FA turnover [5,6]. Growing evidence indicates that autophosphorylated FAK (pY397FAK) is increased in various types of tumor [15-17]. Also, pY397FAK was found in invasive ovarian carcinomas, but not in normal ovarian epithelium [18]. The *in vitro *invasiveness, spread, and migratory abilities of ovarian cancer cell lines are decreased by the introduction of the dominant-negative construct of FAK [11]. These observations suggest that inhibition of FAK activation might be an anti-cancer mechanism [6].

Peroxisome proliferator-activated receptor gamma (PPARγ) is a ligand-activated transcriptional factor and a member of the nuclear hormone receptor superfamily [19]. A number of PPARγ ligands have been identified. Examples include natural prostaglandins, such as 15-deoxy-Δ^12,14^-PGJ2 (15d-PGJ2), and synthetic antidiabetic thiazolidinediones (TZDs), such as troglitazone (TGZ) and ciglitazone (CGZ) [20,21]. TZDs are widely used as antihyperglycemic agents [22]. PPARγ ligands may have potential as anticancer agents [23,24]. TZDs are known to act by inducing mitotic arrest and apoptosis in most cancer cells. Cell apoptosis induced by PPARγ ligands is usually accompanied by cell detachment from the culture substratum [25,26]. The effect of PPARγ ligands on focal adhesions (FAs) assembly has been examined.

PPARγ ligands induce focal adhesion disassembly and decrease in FAK phosphorylation, which may be involved in induction of apoptosis [25,27]. On the other hand, PPARγ ligands has been proposed to inhibit cell motility through its effect on actin organization [28], increase of c-myc expression [29] and inhibition of angiogenesis [30]. In addition, recent reports indicate that PPARγ ligands can reduce human pancreatic cancer cells and myeloid leukemia cells invasion through modulation of the plasminogen activator system or activities of matrix metalloproteinases [28,31-33]. Although these studies imply that PPARγ ligands have potential as anti-metastatic agents, their effects on cancer cell migration remain uninvestigated.

PTEN (phosphatase and tensin homologue deleted on chromosome ten), an identified tumor suppressor, is also involved in the regulation of cell migration [34-37]. Its proposed function involves modulation of FAK phosphorylation [36,37]. Recent study revealed complex regulation of activity of PTEN. The membrane-binding and activation mechanism of PTEN is determined by phosphorylation of its C-terminal tail, and C-terminal phosphorylation suppresses PTEN membrane recruitment [38-40]. Dephosphorylation of PTEN promotes its transfer to the cell membrane and induces its phosphatase activity to suppress growth factor-mediated Akt survival signaling, or interact with phosphorylated FAK to dephosphorylate FAK [36,37,41]. Interestingly, recent studies found that PPARγ ligands can upregulate PTEN expression by enhancing PPARγ transcriptional activity in several types of tumor cell lines [42-44]. However, the influences of PPARγ ligands on ovarian carcinoma of PTEN expression and activity remain unclear.

Cell migration is an important process of metastasis. The effect of PPARγ ligands on local motility, their potential effect on FAs formation, and their anticancer effect on ovarian cancer cells is unclear and not well studied. Therefore, the purpose of this study was to clarify the effect of TGZ on the cell motility of a human ovarian cancer cell line.

## Methods

### Cell culture

Human ovarian carcinoma cell line, ES-2, was obtained from the American Type Culture Collection (ATCC; Rockville, MD, USA) and grown in McCoy's 5A medium with 10% FBS.

### Wound healing assay

Cells (1 × 10^6^) were plated on 6-well culture plates (Corning, Corning, NY, USA) in 10% FBS-containing medium. Upon confluence, the cell layer was scratched with a P-200 pipette tip. The cells were then cultivated in complete medium in the presence or absence of TGZ (Calbiochem, La Jolla, CA, USA). Photographs of the wound adjacent to reference lines scraped on the bottom of the plate were taken using a Nikon ECLIPSE TS100 microscope (under 20× field) at various time points and then counted the number of migrated cells from these photographs.

### Transwell chamber migration assay

The assays were conducted using 8.0-μm pore size and 6.5 mm diameter transwell filters (Costar, Cambridge, MA, USA). The undersurface of the polycarbonate membrane of the chambers was coated with FN (10 μg/ml in PBS; 2 h at 37°C). The membrane was washed in PBS to remove excess ligand, and the lower chamber was filled with 500 μL of 10% FBS-containing medium. ES-2 cells were harvested using limited trypsin treatment by washed twice in medium containing 0.5 mg/ml soybean trypsin inhibitor (Sigma, St. Louis, MO, USA) and once in serum-free medium. Cells (1 × 10^5 ^per chamber) were resuspended in 0.2 ml of serum-free medium and added to the upper chamber for 1 h then treated with TGZ at the concentrations indicated in the text. After 5 h at 37°C in 5% CO_2_, cells were fixed with 4% paraformaldehyde for 15 min at RT, and stained with 0.1 mg/mL crystal violet solution. The cells on the upper surface of the membrane were removed using cotton buds. The number of migrated cells on the underside of the membrane was counted microscopically using a 20× objective (cells/mm^2^).

### Cell spreading assay

ES-2 cells were harvested using limited trypsin treatment as described (above) under Transwell Chamber Migration Assay. Cells (3 × 10^5 ^per 12-well chamber) were resuspended in complete medium with or without 20 μM TGZ for 1 h on human FN-coated coverslips. The mean size of the cells was quantified by analyzing 100 cells from randomly selected fields using MetaMorph software (Universal Imaging, Downingtown, PA, USA).

### Immunocytochemistry

ES-2 cells were harvested using limited trypsin treatment as described (above) under Transwell Chamber Migration Assay. Cells were plated on human FN-coated coverslips in complete medium contained DMSO or 20 μM TGZ for 4 h or 24 h, fixed with 4% paraformaldehyde (20 min), permeabilized by 0.5% Triton X-100 in PBS (20 min), and blocked with 1% bovine serum albumin (1 h). Cells were incubated with monoclonal antibodies to paxillin (1:200 dilution; BD Transduction Labs, Lexington, KY, USA), vinculin (1:200; Chemicon, Temecula, CA, USA), or PTEN (1:200; Santa Cruz Biotechnology, Santa Cruz, CA, USA), and then with FITC-labeled horse anti-mouse IgG (1:500; Vector Labs, Burlingame, CA, USA). Cytoskeletal proteins were visualized by 30 μM rhodamine-phalloidin (Sigma). After final washes and mounting, cells were examined using confocal microscopy (LSM410, Carl Zeiss, Oberkochen, Germany).

### Cell lysis, fractionation, and SDS-PAGE

Following treatment, ES-2 cells were scraped into lysis buffer (150 μL/35 mm well) containing 20 mM HEPES (pH 7.4), 1% SDS, 150 mM NaCl, 1 mM EGTA, 5 mM β-glycerophosphate, 10 mM sodium pyrophosphate, 10 mM sodium fluoride, 100 μM sodium orthovanadate, 10 μg/mL leupeptin, and 10 μg/mL aprotinin. The lysate was incubated on ice for 15 min. Cell debris was removed by centrifugation at 12,000 × g for 15 min at 4°C. Membrane and cytoplasmic proteins from ES-2 cells were extracted with ProteoExtract^® ^Native Membrane Protein Extraction Kit (Calbiochem) according to the manufacturer's instructions. Protein concentration of each sample was assayed using BCA Protein Assay Reagent according to manufacturer's instructions (Pierce Biotechnology, Rockford, IL, USA). Each cellular fraction was then resolved on a 12% SDS-polyacrylamide gel electrophoresis (PAGE) and then electrotransferred to polyvinylidene fluoride (PVDF) membranes (Immobilon-P; Millipore, Bedford, MA, USA).

### Immunoprecipitation and Western blot analysis

Immunoprecipitation was performed using anti-FAK (4 μg/mL; BD Transduction Laboratories) antibody. Immunoprecipitates were resolved by SDS-PAGE, and analyzed by Western blotting with anti-phospho-tyrosine antibodies (1:1000 dilution; Abcam Ltd, Cambridge, UK) as described previously [45]. For Western blot analysis, fifty micrograms of each protein sample was subjected to SDS-PAGE and electrotransferred to PVDF membranes, blocked, and then incubated with primary antibody. Proteins of interest were detected using appropriate IgG-HRP secondary antibody (Santa Cruz Biotechnology, Santa Cruz, CA, USA) and ECL reagent (Amersham, Arlington Heights, IL, USA). Primary antibodies included phospho-Tyr-397 FAK (1:1000; Biosource, Camarillo, CA, USA), FAK (1:1000; BD Transduction Laboratories), PTEN (phospho S380 + T382 + T383; 1:1000; Abcam Ltd), PTEN (1:1000; Santa Cruz Biotechnology), PPARγ (1:1000 dilution, Santa Cruz Biotechnology), Bax (1:1000, Upstate Biotechnology, Lake Placid, NY, USA), pS473 Akt (1:1000; Promega, Madison, WI, USA), Akt (1:1000; Santa Cruz Biotechnology), N-cadherin (1:1000; Transduction Laboratories), and β-actin (Sigma) and were used according to the manufacturers' instructions. X-ray films were scanned on the Model GS-700 Imaging Densitometer (Bio-Rad Laboratories, Inc., Hercules, CA, USA) and analyzed using Labworks 4.0 software.

### PPARγ small interfering RNA treatment

The sequences of PPARγ1 siRNA and control pGL3 siRNA duplexes were synthesized (Dharmacon, Lafayette, CO, USA) as previously described [46]. For the transfection procedure, cells were grown to 70% confluence, and PPARγ siRNA or control siRNA was transfected using Oligofectamine (Invitrogen, Carlsbad, CA, USA). The final concentration of siRNA was 50 nM. Six hours after siRNA transfection, cells were resuspended in new culture media, incubated for additional 16 h, and then treated with TGZ.

### Statistical analysis

Data are expressed as mean ± standard deviation (SD) of three independent experiments, each done in triplicate (n = 3~4 dishes). The Mann-Whitney *U *test was used to determine statistically significant differences. *P *values < 0.05 were considered significant.

## Results

### TGZ dose- and time-dependently inhibits ES-2 cell migration

**Figure 1:**
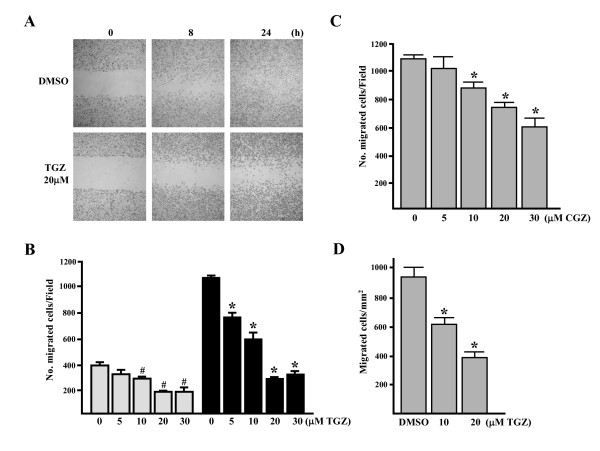
(A and B) TGZ inhibits ES-2 cell migration in a wound healing assay. Cells were wounded and then treated with vehicle (DMSO) or TGZ (5–30 μM) for 8 h (grey column) and 24 h (black column) in 10% FBS-containing medium. At 0, 8 and 24 h, phase-contrast pictures of the wounds at three different locations were taken and then migrated cells in the wound were counted. ^#^*P *< 0.05 *vs*. 8 h untreated cells and *P < 0.05 *vs*. 24 h untreated cells. (C) CGZ inhibits ES-2 cell migration in a wound healing assay. Cells were treated with various concentrations of CGZ (5–30 μM) and then the migrated cells were counted after treatment for 24 h. *P < 0.05 *vs*. control. (D) TGZ inhibits migration of ES-2 cells in a transwell assay. ES-2 cells (1 × 10^5^) were treated with vehicle (DMSO) or TGZ (10 or 20 μM) for 5 h at 37°C and cell motility was determined as described in Materials and Methods. *P < 0.001 *vs*. DMSO treatment.

Cells of the ovarian carcinoma cell line, ES-2, were exposed to increasing concentrations of TGZ (5–30 μM) in 10% serum-containing medium, and cell migration was examined using the *in vitro *wound healing assay. Figure 1A shows representative photographs of cells migrating into scratch wounds. The number of cells migrating into the wound decreased in a TGZ concentration-dependent manner (Figure 1B). At 20 μM, TGZ decreased this number 48 ± 2% and 76 ± 3% at 8 h and 24 h, respectively. The decrease did not appear to be due to cytotoxicity since TGZ (5~20 μM) showed no inhibitory effect on cell proliferation as determined by MTT assay (data not shown). In addition, CGZ treatment for 24 h also blocked ES-2 migration, albeit to a lesser degree (Figure 1C compared with 1B).

In an *in vitro *transwell migration assay, TGZ reduced ES-2 cell migration to the bottom chamber containing fetal bovine serum (FBS) by ~1.5- and ~2.4-fold at 10 μM and 20 μM, respectively (compared with DMSO treated cells; *P *< 0.001, Figure 1D). These results indicate that, at non-toxic levels, TGZ and CGZ are capable of inhibiting the migration of ES-2 cells.

### TGZ inhibits stress fibers and focal adhesion formation

**Figure 2:**
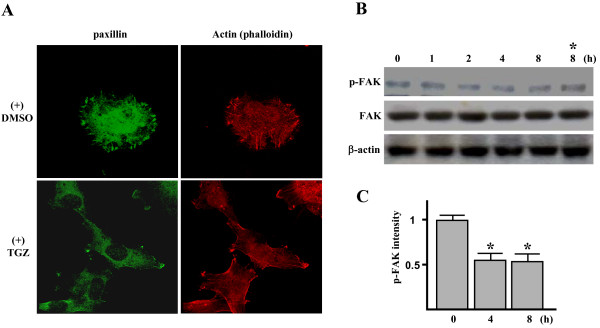
**TGZ inhibits stress fiber and focal adhesion formation**. (A) Effect of TGZ on the distribution of paxillin and stress fibers. ES-2 cells were plated on FN (10 μg/mL)-coated coverslips and incubated in 10% FBS medium with 0.1% DMSO or 20 μM TGZ for 4 h. Cells were then double stained with rhodamine-labeled phalloidin and antibodies to paxillin (FITC). These results are representative of triplicate experiments. (B) Time-course study of the tyrosine phosphorylation level of focal adhesion kinase (FAK) in TGZ-treated ES-2 cells. ES-2 cells were treated with 20 μM TGZ for different intervals. The cell homogenates were immunoprecipitated from 1 mg of total cellular protein by anti-FAK antibody and subjected to Western blot analysis with anti-phosphotyrosine antibody (p-FAK) or anti-FAK antibody (FAK). The cell homogenates were also analyzed for β-actin levels by Western blotting as indicated. Immunoblot results are from a representative experiment performed in triplicate with β-actin as loading control. Symbol (*) indicates cells that were treated with DMSO for 8 h.

We next examined the effect of TGZ on focal adhesions (FAs) formation because its impairment has been shown to reduce cell migration [6]. After four hours of adhesion to fibronectin (FN)-coated surfaces, ES-2 cells formed numerous FAs, which were stained by anti-paxillin. In cells incubated with 20 μM TGZ, FAs were substantially reduced (Figure 2A). Similar inhibitory effect of TGZ was observed when FAs formation was identified by anti-vinculin antibody (data not shown). Reduction in the number of FAs was accompanied by an overall decrease in actin stress fiber (Figure 2A). It is highly possible that TGZ treatment decreases the transport or maintenance of cytoskeletal proteins in FAs, such as paxillin and vinculin, thereby reducing the number of complexes available for the formation of strong focal points and actin bundling.

Since tyrosine phosphorylation of FAK (p-FAK) regulates FAs formation, the level of tyrosine phosphorylation of FAK after treatment with 20 μM TGZ was investigated. Western blot analysis revealed a one-half-fold reduction in p-FAK level after treatment for 4 h and 8 h. The total level of FAK, however, was not changed (Figure 2B and 2C).

### Effects of TGZ on cell spreading

**Figure 3:**
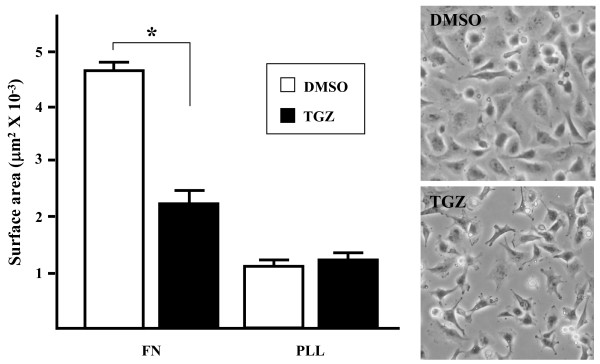
**TGZ inhibition of cell spreading**. ES-2 cells treated with DMSO or 20 μM TGZ were plated on coverslips which were coated with FN (10 μg/ml) or poly-L-lysine (25 μg/ml, PLL). The cell surface area after 1 h was determined using MetaMorph software  on at least 100 cells from five different fields (100 × magnification); *n *= 3 experiments. *P < 0.005 *versus *control. Right panels represent phase-contrast photographs of ES-2 cells treated with DMSO or TGZ for 1 h.

To further assess TGZ inhibition of FA formation, cell spreading on FN was examined. Within 1 h after seeding, ES-2 cells, untreated or treated with DMSO, spread well on FN-coated coverslips. However, treatment with 20 μM TGZ led to cell retraction from the substratum, loss of contacts between neighboring cells, and eventually in a spindle-shaped morphology (Figure 3, pictures). The rounded morphology of cells grown on poly-L-lysine (PLL) was not affected by TGZ treatment (data not shown). Quantitative morphometric analysis (Materials and Methods) revealed that TGZ treatment reduced the surface area of spread cells by twofold (Figure 3, bar graph, *P *< 0.005). Thus, TGZ appears to interfere with the molecular events that drive spreading and the extension of membrane protrusions. In addition, the effect of TGZ seems to be integrin-dependent since cell spreading on PLL-coated plates was not affected.

### The TGZ-mediated anti-migratory effect is independent of the induction of PPARγ expression

**Figure 4:**
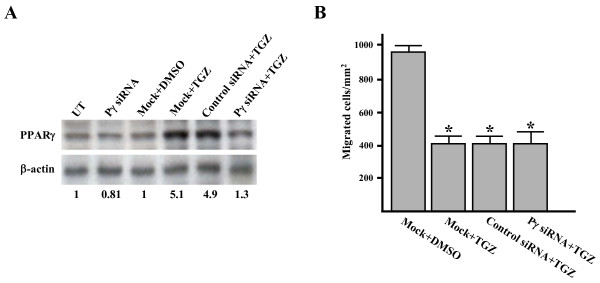
**The TGZ-induced anti-migratory effect is independent of PPARγ expression**. (A) ES-2 cells were either left untreated (UT) or transfected with PPARγ (Pγ) siRNA or control siRNA (50 nM each) as described in Materials and Methods, then treated with or without 20 μM TGZ for an additional 5 h. Western blot analysis of the cell lysates was carried out to determine the levels of PPARγ. Bottom of PPARγ panel indicates the quantity of PPARγ normalized to β-actin. All blots shown are one of three experiments. (B) Transwell assay of siRNA-transfected ES-2 cells. Mock or siRNA-transfected ES-2 cells were treated with TGZ for 5 h and cell migration quantified by Transwell assay as described in Figure 1 *d*. "Mock" indicates cells that were treated with transfection reagent. *P < 0.05 *vs*. DMSO-treated cells.

Our previous report (Ref. [48]) and this study (Figure 4A) indicate that TGZ can enhance PPARγ expression in ES-2 cells. To determine whether TGZ-induced PPARγ expression is required for inhibition of cell migration, ES-2 cells were transfected with PPARγ-specific siRNA and then assayed for PPARγ expression and cell migration in the presence of 20 μM TGZ. Western blotting showed that the basal level of PPARγ protein was only slightly decreased by the PPARγ siRNA transfection. However, TGZ-induced PPARγ expression was significantly reduced by the PPARγ siRNA and not by the control siRNA (Figure 4A). The transwell cell migration assay revealed the similar capacity of TGZ to inhibit the migration of both PPARγ siRNA- and control siRNA-transfected cells (Figure 4B). Therefore, TGZ-mediated inhibition of ES-2 cell migration is through a PPARγ-independent mechanism.

### TGZ dose- and time-dependently inhibits FAK autophosphorylation and PTEN phosphorylation

**Figure 5:**
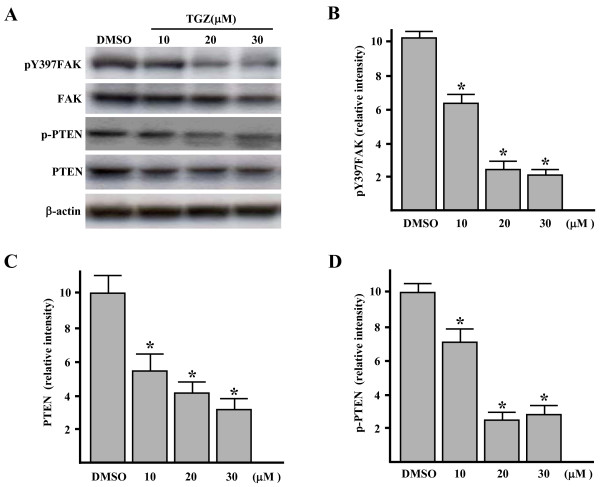
**Dose study of the effect of TGZ on the phosphorylation and expression of FAK and PTEN**. ES-2 cells were treated with different doses of TGZ for 24 h (A). The cell homogenates were subjected to Western blot analysis with various antibodies as indicated. Immunoblot results are from a representative experiment performed in triplicate with β-actin as loading control. (B) After densitometric scans of triplicate blots, values for pY397FAK were normalized to total FAK. (C and D) Values for p-PTEN and PTEN were normalized to β-actin. *P < 0.05 *vs*. DMSO-treated cells.

To investigate whether the FAK autophosphorylation (pY397FAK) could be affected after TGZ treatment, Western blot analysis was performed and revealed that exposure of cells to varying concentrations of TGZ (10, 20 and 30 μM, 24 h) induced a concentration-dependent decrease in pY397FAK level (Figure 5A and 5B, *P *< 0.05 *vs*. DMSO-treated cells). Thus, TGZ may inhibit migration by dose-dependently preventing FAK autophosphorylation.

Accumulating evidence indicates that PTEN inhibits cell migration, possibly by dephosphorylating p-FAK at Y397 [36,37]. The reduction in pY397FAK level raises the prospect that PTEN is involved in TGZ inhibition of migration. We thus studied the levels of PTEN and phosphorylated PTEN in TGZ treated cells and found TGZ (10–30 μM) reduced PTEN level (~1.8–3.1 fold lower than DMSO-treated cells; Figure 5A and 5C). Interestingly, the phosphorylated PTEN (p-PTEN) level was also dose-dependently decreased (Figure 5A and 5D, *P *< 0.05). This suggests that TGZ treatment induces PTEN degradation and PTEN dephosphorylation in a dose-dependent way.

**Figure 6:**
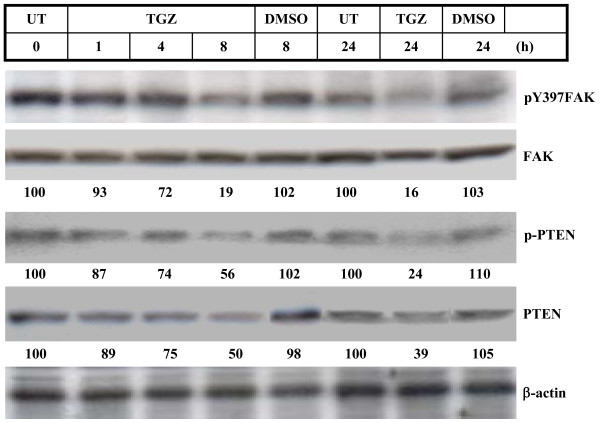
**Time-course study of the effect of TGZ on the phosphorylation and expression of FAK and PTEN**. ES-2 cells were treated with 20 μM TGZ for the time intervals indicated. The cell homogenates were subjected to Western blot analysis with various antibodies as indicated. Immunoblot results are from a representative experiment performed in triplicate with β-actin as loading control.

To further establish the interaction between PTEN and pY397FAK, we investigated FAK and PTEN levels at various times after TGZ exposure. Western blot analysis indicated that exposure of cells to 20 μM TGZ for 8 h and 24 h caused dramatic decreases in the levels of both pY397FAK and p-PTEN as compared with untreated- or DMSO-treated cells (Figure 6). In addition, total PTEN level was markedly decreased upon TGZ treatment for 8 h and 24 h. This indicated that TGZ can induce a time-dependent reduction in pY397FAK, p-PTEN, and PTEN levels.

### TGZ treatment causes plasma membrane accumulation of PTEN

**Figure 7:**
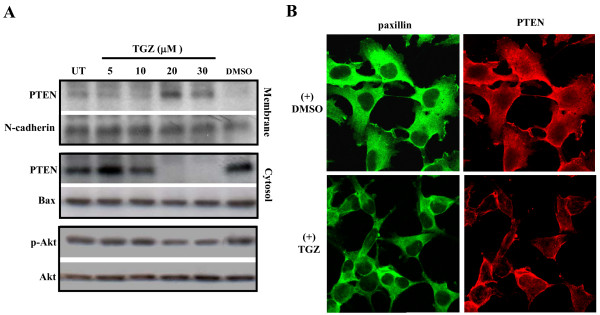
**TGZ changes the ratio of PTEN localized in cytosol and plasma membrane**. (A) ES-2 cells were treated with various concentrations of TGZ for 24 h. Cell membrane and cytosolic fractions were isolated as described in Materials and Methods and then subjected to SDS-PAGE and Western blot analysis. The result of one representative assay from two similar independent experiments is shown. (B) Effect of TGZ on the distribution of PTEN. ES-2 cells were plated on FN (10 μg/mL)-coated coverslips and incubated in 10% FBS medium with 0.1% DMSO or 20 μM TGZ for 24 h. Cells were then double stained with paxillin (FITC) and PTEN (rhodamine). These results are representative of triplicate experiments.

Dephosphorylation of the C-terminal of PTEN induces the lipid phosphatase activity of PTEN which then leads to accumulation of PTEN on cell membrane [36,37,40]. The PTEN dephosphorylation not only brings PTEN to its substrate but also leads to the degradation of PTEN itself [47]. The degradation of PTEN after TGZ treatment (shown above) led us to suspect that PTEN is activated by TGZ. To confirm this, PTEN accumulation on the cell membrane was assayed. Cell membrane and cytosolic fractions of TGZ-treated cells were isolated for Western blot analysis. As shown in Figure 7A, level of PTEN in the membrane fraction was not significantly increased in cells treated with 5 μM and 10 μM TGZ as compared with untreated or DMSO-treated cells and most PTEN were detected in the cytosolic fraction. However, at 20 μM and 30 μM, TGZ triggered PTEN association with the cell membrane and PTEN was barely detected in the cytosolic fraction. N-cadherin and Bax were used as respective markers of cell membrane protein and non-membrane protein. Thus, PTEN membrane translocation is induced by high concentration of TGZ.

To further confirm that cell membrane accumulation of PTEN can be induced by high concentration of TGZ, immunofluorescence analysis of PTEN localization was performed. As shown in Figure 7B, in control cells, PTEN protein was in cytoplasm and cell membrane and had a similar distribution to paxillin. However, after treatment with 20 μM TGZ for 24 h, membrane accumulation of PTEN protein increased, suggesting that the higher concentration causes cell membrane accumulation of PTEN, which is a sign of PTEN activation. This supports the notion that TGZ induces FAK dephosphorylation by activating PTEN.

Translocation of activated PTEN to the cell membrane leads to the dephosphorylation of Akt [41]. Since TGZ treatment leads to membrane accumulation of PTEN, it was of interest to document the activation of PTEN by TGZ. Levels of Akt phosphorylation (p-Akt) were moderately decreased ~2–3-fold in TGZ (20 and 30 μM) treated cells as compared to TGZ (5 and 10 μM) or DMSO-treated cells; while total Akt level remained unchanged (Figure 7A). This suggests that the PTEN accumulated on cell membrane is activated, or at least its ability to inhibit phosphorylation of Akt is functional. It is plausible to assume that activated PTEN is also responsible for the dephosphorylation of pY397FAK.

## Discussion

We recently showed that PPARγ agonists (TGZ and CGZ; 1–50 μM) dose-dependently induced growth arrest and inhibited the viability of ES-2 cells after long-term (48–72 h) treatment [48]. Here we further provide *in vitro *evidence for additional antitumor properties of TGZ by showing its potential effect on cell spreading and migration. We found that TGZ time- and dose-dependently inhibits 1) cell migration before cell growth and cell viability and 2) FAK autophosphorylation, which correlates with inhibition of cell migration. In addition, we found that TGZ treatment at higher concentrations (20 and 30 μM) caused cell membrane accumulation of PTEN, suggesting the involvement of PTEN in TGZ's inhibitory effect on FAK activation and cell migration. Our data suggest that TGZ may possess anti-metastatic in addition to anti-proliferation effects.

In the scratch wound-healing assay, the ES-2 cell line (compared with certain other ovarian carcinoma cell lines) has a higher degree of motility, suggesting it can be a suitable model system to study cell migration *in vitro *[49]. In the present study, we used the scratch wound-healing assay to show TGZ (5–30 μM) can impair ES-2 cell migration in a time- and dose-dependent manner (Figure 1A and 1B). Previous reports that antibodies against fibronectin (FN) can partially inhibit ovarian carcinoma cell motility and reduce intraperitoneal spread imply that the interaction of substratum FN with cell integrins is a determinant of migration of ovarian carcinoma cells [50-52]. Our results further clarified TGZ's anti-migratory effect on ES-2 cells grown on FN substratum by showing that spreading (Figure 3), F-actin/FAs formation (Figure 2A), and transwell chamber migration (Figure 1D) were all markedly inhibited by TGZ treatment. Such findings suggest that TGZ might have anti-migratory activity against ovarian carcinoma cells.

Although thiazolidinedione ligands (TZDs) were initially identified as ligands of PPARγ, accumulating evidence indicates that TGZ can affect cell function independent of PPARγ receptor activation. Previously, TGZ was shown to induce cancer cell apoptosis by non-PPARγ mechanisms [21,53]. In our study, both PPARγ-specific siRNA (Figure 4) and a selective PPARγ antagonist, GW9662 pretreatment (10~20 μM, 2 h; our unpublished data) failed to reverse the inhibition of migration, indicating for the first time that TGZ inhibits migration through a PPARγ-independent mechanism.

*In vitro *studies reveal that FAK kinase activity is essential for cell migration [5,6]. Moreover, the pY397FAK localization assayed by immunohistochemistry is detected only in invasive ovarian carcinomas but not normal ovarian tissue specimens [18]. FAK activation is initiated by autophosphorylation at Tyr 397 (pY397FAK) [12]. Our results reveal that TGZ dosages required to inhibit cell migration (Figure 1B and 1D) are the same as those required to decrease pY397FAK level (Figure 5B), suggesting pY397FAK may be a potential therapeutic target for treating ovarian carcinoma metastasis. Interestingly, ES-2 cells treated with 20 μM TGZ for 4–8 h caused ~50% reduction of the overall levels of tyrosine phosphorylation of FAK (p-FAK) (Figure 2B and 2C), whereas same treatment for 8 h caused more dramatic reduction of pY397FAK (~80% reduction; Figure 6). These suggest that pY397FAK may be a major target of TGZ treatment. The more dramatic inhibition of pY397FAK than p-FAK was also observed previously in hypoxia-induced cytotrophoblast migration *in vitro *[54].

The underlying mechanism of TGZ suppression of FAK autophosphorylation remains unclear. pY397FAK levels are increased by integrin clustering and FAK-Src kinases association [9,12,13]. Decrease of pY397FAK levels by TGZ may be exerted through interference with the interaction between FAK and its activating molecules. In addition, FAK activity is regulated by protein-tyrosine phosphatases (PTPs)-mediated dephosphorylation. FAK is dephosphorylated by several PTPs such as SH2-containing tyrosine phosphatase 2 (SHP2) and low molecular weight tyrosine phosphatase (LMW-PTP) or PTEN [34,35,55-57]. These are candidate mediators of the TGZ effect. The mechanisms involved in the inhibition of FAK autophosphorylation by TGZ will be important to identify in future research.

Among the PTPs, PTEN particularly interests us since PTEN has been linked to regulation of cell migration [36,37]. The activity of PTEN itself is under novel regulation. It is widely accepted that the lipid phosphatase of PTEN is activated with its dephosphorylation [38-40]. The dephosphorylated PTEN is preferentially translocated to the vicinity of its substrates in the cell membrane such as pY397FAK [58]. The dephosphorylation of PTEN also leads to its eventual degradation [47]. The activation of PTEN by TGZ is suggested by several of our observations including: 1) decrease in phosphorylated AKT, which indicates activation of PTEN (Figure 7A); 2) dose-dependent decrease in p-PTEN levels accompanied by decrease in total PTEN (Figure 5C and 5D); and 3) PTEN membrane translocation (Figure 7). These lead to the suggestion that TGZ can suppress pY397FAK levels through PTEN activation. In addition, we suspect at least two mechanisms involving TGZ regulation of pY397FAK, since at 10 μM, TGZ treatment inhibited pY397FAK but not PTEN translocation. Thus TGZ at 10–30 μM may affect FAK autophosphorylation by blocking its interaction with activating proteins. At higher than 10 μM, TGZ leads to increased PTEN activity and to the further inhibition of pY397FAK formation.

The mechanism of TGZ-induced PTEN dephosphorylation/activation in ES-2 cells remains unclear. The activity of PTEN is regulated by casein kinase 2 (CK-2) and small GTPase RhoA and its downstream effector, RhoA-associated kinase (Rock) [47,59]. Interestingly, inhibition of CK-2 and PTEN activity by TGZ was recently observed in endothelial cells [60]. The possible role of CK-2 and RhoA/Rock on TGZ-caused PTEN dephosphorylation can be tested experimentally and deserves further investigation.

## Conclusion

TGZ treatment can impair spreading and migration of ES-2 cells, an ovarian carcinoma cell line with known high motility. We suggest that TGZ or its analogues may be new drugs for the prevention and treatment of ovarian cancer.

## Competing interests

The author(s) declare that they have no competing interests.

## Authors' contributions

YPT and YCY conceived the study, participated in the study design, and manuscript preparation. TCH and SLC participated in the study design, performed data analysis, aided in troubleshooting assays and drafted the manuscript. TCH, HYL and JYW conducted all experiments in this paper. All authors read and approved the final manuscript.

## Pre-publication history

The pre-publication history for this paper can be accessed here:


